# MASSIVE HAEMOTHORAX IN ASYMPTOMATIC PSEUDOCYST PANCREAS

**DOI:** 10.4103/0970-2113.59594

**Published:** 2008

**Authors:** Ramakant Dixit, Sidharth Sharma, Lokendra Dave

**Affiliations:** Deptt. of Respiratory Medicine & Tuberculosis, J.L.N. Medical College, Ajmer-305 001, Rajasthan, India

**Keywords:** Haemothorax, Pseudocyst pancreas

## Abstract

The case of a 35-year old man who presented with massive left sided haemothorax as a complication of an asymtomatic pancreatic pseudocyst is descibed. The diagnosis was confirmed by very high amylase content of the pleural fluid. The complications of pancreatitis and pancreatic pseudocyst are also briefly discussed. Haemothorax represents an unusual pulmonary complication of pseudocyst pancreas and should be considered in the differential diagnosis of pleural fluid collection in pancreatitis.

## INTRODUCTION

The development of pleural effusion is a common manifestation of pulmonary diseases. In about half of all cases of pleural effussion, the diagnosis is usually apparent after a thorough history and physical examination and a workup, including thoracocentesis and other selected diagnostic tests^1^. The remaining pleural effusions are more difficult to diagnose. Unfortunately, as many as 15-20% cases of all pleural effusions remain undiagnosed despite intensive efforts^2^.

An unusual and over-looked cause of pleural effusion is an intra abdominal process. Pleural effusion is an uncommon complication of pancreatitis. It is often left sided and associated with acute pancreatitis^3^. Development of massive left sided pleural effusion in form of haemothrax in a patient with no clinical evidence of pancreatic disease is very rare. We report such a case here.

## CASE REPORT

A 35-year old man was admitted with chief complaints of left sided chest pain, increasing shortness of breath over last two week and fever with vomiting for last three days. He denied any cough, expectoration, haemoptysis, abdominal discomfort (acute/chronic, pain), weight loss or trauma in recent or remote past. He was not taking any medications prior to the presentation.

On physical examination, patient appeared pale and in distress (respiratory rate 32/min.). Clinical examination of chest revealed dull percussion note and diminished breath sound all over left hemi thorax with shift of heart and trachea towards right side. A provisional diagnosis of massive left sided pleural effusion with mediastinal shift was made and confirmed by chest radiography. A closed tube thoracostomy was performed on left side and about three liters of grossly haemorrhagic fluid was drained, resulting in relief from distress. The routine investigations of blood and urine, apart from blood biochemistry (renal function tests, liver function tests, lipid profile and electrolytes etc.) were within normal limits. The pleural fluid was almost bloody with a protein of 5.6 gm%, LDH 6400, RBC 3.4×10^6^/mm^3^, WBC 21,000/mm^3^ (85% polymorphs and 15% lymphocytes) and ADA level 15 U/L. No organisms were identified on Gram's or Ziehl Neelson staining of pleural fluid smear and subsequent culture was also sterile. Pleural fluid cytology was negative for malignant cells and pleural biopsy showed normal pleura. The haematocrit of pleural fluid was 33% in comparison to venous blood sample of 35%, thus confirming the haemothorax. To rule out a possible intrathoracic vascular pathology/bleed causing massive left sided haemothorax, a computed tomography (CT) scan was performed which revealed massive collection in left hemi thorax with thick enhancing pleura at periphery (split pleura sign) and air fluid levels. The underlying lung was partially collapsed with crowding of vessels. No cardiovascular abnormality was detected. Simultaneous study of abdominal structures incidentally disclosed a well-defined thick walled cystic lesion in the lessor sac, medial to stomach and superior to tail of pancreas suggestive of pseudocyst pancreas, which was extending upward. Other abdominal structures including pancreas and major intra abdominal vessels appeared normal.

With this unexpected CT scan finding in the abdomen, a possibility of pleural fluid collection secondary to pseudocyst pancreas was considered and confirmed by subsequent pleural fluid amylase level, which was 25,000 Units. The serum amylase level was also raised (18,500 units). Patient was given intravenous fluids, antibiotics and blood transfusion. Before a surgical repair could be done, patient died suddenly possibly due to aspiration following an episode of vomiting.

## DISCUSSION

Pleural effusion though uncommon, is well recognized in patients with acute pancreatitis, when the diagnosis is usually obvious. Ascites or lesser sac fluid collection in chronic pancreatic disease may also be associated with pleural effusion. Pancreatic disease is generally not considered in patients presenting solely with massive haemorrhagic pleural effusion^4^. Various mechanisms responsible for pleural effusion in pancreatitis includes direct contact of pancreatic enzymes with the diaphragm, haematogenous transfer of pancreatic enzymes into the pleura, communication between the pleural and peritoneal cavities through the trans-diaphragmatic lymphatic and in rare cases direct communication of the pseudocyst with the pleural cavity^5^. The enzyme-rich pleural effusions represent a disruption of the dorsal pancreatic ductal system^6^.

Pancreatic pseudocyst is well-recognized complication of non-traumatic pancreatitis. They are said to follow a bout of acute pancreatitis by three to four weeks and most often manifested by an epigastric mass or sensation of fullness. Bleeding, extension, fistulisation and infection are known complications of pseudocyst^7^. Bleeding originating in pancreatic pseudocysts manifested by circulatory collapse has been reported in cases of spontaneous rupture into the colon, peritoneal cavity and stomach^8^. Extension of the cyst may occur into the posterior mediastinum, neck and left pleural space^9^. The cyst fistulising and bleeding massively amounting to cause haemothorax has been very rarely reported^7^, however that too occurred in a patient with clinical background of acute pancreatitis. The case presented here seems unusual in that the patient did not have symptoms and signs to suggest pancreatic disease. He did not drink alcohol and had no symptoms to suggest gall bladder or pancreatic disease in past and presented with massive haemothorax on left side secondary to asymptomatic accidentally detected pseudocyst pancreas on CT scan. The diagnosis was confirmed by very high amylase content of pleural fluid.

Although massive haemorrhagic pleural effusion with a high pleural fluid amylase^4^,^10^ has been reported but pleural fluid in which the haematocrit is equal to or very near the systemic venous haematocrit is reported only once^7^. The present report is probably first one on the occurrence of massive left sided haemothorax secondary to asymptomatic pseudocyst in the absence of any features/background of pancreatitis.

Currently, treatment of patients with pseudocyst pancreas is based on the clinical setting, the presence or absence of symptoms, the age and size of the pseudocyst and the presence or absence of complications”. Endoscopic retrograde cholangiopancreatography (ERCP) and magnetic resonance cholangiopancreatography (MRCP) are useful diagnostic modalities in recurrent or multiple pseudocyst and are highly accurate in depicting pancreatic ductal anatomy before operative intervention. ERCP also provides opportunity to simultaneously direct endotherapy toward decompressing the pancreatic fluid collection and helps in placement of stent when there is pancreatic ductal disruption^6^,^12^.

Pancreatic pleural effusion, results from pancreatic ductal disruption posteriorly with fluid draining through reteroperitonium to the pleural space. Non-operative treatment is initially indicated in patients with pancreatic pleural effusion: The therapeutic rationale is to decrease pancreatic exocrine secretion and thereby to encourage the pancreatic ductal disruption to seal. Management includes the prohibition of oral feeding, total parenteral nutrition, and thoracocentesis. The long acting somatostatin analogue octreotide may be of benefit in some cases. Non-operative management is recommended for a 2 to 3 week period and may resolve the clinical entity in 50 to 60% of patients. In patients not cured by non-operative management, surgical therapy is indicated after delineation of pancreatic ductal anatomy using endoscopic retrograde pancreatography. Endoscopic therapy with sphincterotomy or pancreatic duct stenting has been reported to assist healing in smaller number of patients, and it may be tried if initial conservative therapy fails to seal the duct leak. Distal pancreatic duct disruption or a leaking pseudocyst in the body or tail of pancreas may be treated with distal pancreatectomy or with Roux-en-Y pancreatico jejunostomy to the leak site. Pancreatic duct leaks in the more proximal aspect of the gland are treated with Roux-en-Y pancreaticojejunostomy to the site of disruption^11^.

The purpose of communicating this case is three folds- (i) in left sided exudative pleural effusion with or without history of acute/ chronic pain abdomen and alcohol intake, amylase activity in pleural fluid should be measured so as to avoid undue delay in diagnosis, repeated thoracocentesis, pleural biopsies, other specific tests and wrong empirical antituberculosis treatment specially in countries like ours; (ii) in undiagnosed pleural effusions, an abdominal cause must always be considered and should be ruled out with CT scan or other requisite studies i.e. Ultrasound to rule out such causes; and (iii) haemothorax should be included in the differential diagnosis of pleural fluid collections in pancreatitis associated with pseudocyst. The bleeding from pseudocyst can be massive enough to warrant transfusion and placement of a chest tube.
